# Synthesis of a Lignin/Zinc Oxide Hybrid Nanoparticles System and Its Application by Nano-Priming in Maize

**DOI:** 10.3390/nano12030568

**Published:** 2022-02-07

**Authors:** Daniele Del Buono, Francesca Luzi, Ciro Tolisano, Debora Puglia, Alessandro Di Michele

**Affiliations:** 1 Dipartimento di Scienze Agrarie, Alimentari e Ambientali, University of Perugia, Borgo XX Giugno 74, 06121 Perugia, Italy; daniele.delbuono@unipg.it (D.D.B.); ciro.tolisano@studenti.unipg.it (C.T.); 2 Department of Materials, Environmental Sciences and Urban Planning (SIMAU), Polytechnic University of Marche, Via Brecce Bianche 12, 60131 Ancona, Italy; f.luzi@staff.univpm.it; 3 Department of Civil and Environmental Engineering, University of Perugia, Strada di Pentima 4, 05100 Terni, Italy; 4 Department of Physics and Geology, University of Perugia, Via Elce di Sotto, 06123 Perugia, Italy

**Keywords:** nanotechnology, lignin nanoparticles, zinc oxide, hybrid materials, seed-priming, plant growth, antioxidant activity

## Abstract

Nanotechnologies are attracting attention in various scientific fields for their technological and application potential, including their use as bio-activators and nanocarriers in agriculture. This work aimed to synthesize a hybrid material (ZnO@LNP) consisting of lignin nanoparticles containing zinc oxide (4 wt %). The synthesized ZnO hybrid material showed catalytic effect toward thermal degradation, as evidenced by the TGA investigation, while both spectroscopic and contact angle measurements confirmed a modification of surface hydrophilicity for the lignin nanoparticles due to the presence of hydrophobic zinc oxide. In addition, the antioxidant activity of the ZnO@LNP and the zinc release of this material were evaluated. At the application level, this study proposes for the first time the use of such a hybrid system to prime maize seeds by exploiting the release characteristics of this material. Concerning the dosage applied, ZnO@LNP promoted inductive effects on the early stages of seed development and plant growth and biomass development of young seedlings. In particular, the ZnO@LNP stimulated, in the primed seeds, a higher content of chlorophyll, carotenoids, anthocyanins, total phenols, and a better antioxidant activity, as supported by the lower levels of lipid peroxidation found when compared to the control samples.

## 1. Introduction

The current context requires urgent measures to offer alternative solutions to reduce or avoid the abuse and over-use of natural resources, as their availability is limited [1]. Furthermore, many different anthropogenic activities result in high impact and pressure on natural resources [2]. In this regard, nanotechnologies are gaining a primary role as they offer a wide range of new nanomaterials, paying particular attention to developing new bio-based solutions [3].

Nanomaterials can be obtained applying conventional procedures or, as it is taking place more recently, green synthetic approaches [4]. The former can be generally characterized by a high environmental impact or energy consumption; the latter usually does not impact or use or produce hazardous by-products and have a very affordable cost [3].

Given the nanomaterials’ numerous potentialities, the most recent research has, on the one hand, shifted its attention toward biomasses of natural origin, exploring many different ways to valorize these frequently ignored resources [1,5]. Furthermore, the research has started combining different nanomaterials, obtaining hybrid, composite, or coated systems, which offers the possibility of effectively modulating the properties of the resulting nanoparticles [6,7]. This last aspect deserves particular attention, as it could offer the possibility to obtain materials that potentiate, modulate, or change particular properties of one or more substances included in the obtained systems [7].

In this context, lignin represents intriguing biomass and a versatile macromolecule, which can be easily scalable to nanostructured forms [8,9]. On the planet, lignin is the second most abundant biomass after cellulose and shows a considerable structural variability depending on the botanical origin and characteristics of the species it derives [9,10]. From a structural point of view, lignin shows three central structural phenolic units, and their relative amounts depend on the plant botanical origin [9]. These main structural phenolic motives are indicated as guaiacyl (G), p-hydroxyphenyl (H), and syringyl (S) units [9,11].

Depending on the application, lignin often undergoes chemical reactions that can modify its molecular structure and insert new functional groups, thus opening the perspective of modulating its properties according to the desired results [12]. Nonetheless, only a very small part of the lignin produced as an agricultural or industrial by-product is recycled for some applications [13]. Most of this biomass and the materials derived are disposed of as waste. As a consequence, this can also create environmental issues [13]. Therefore, lignin properties and the possibility to convert and valorize it obtaining interesting technological materials pose more than one question on the opportunity to pay more effort to the recovery and the sustainable valorization of this biomass.

Concerning zinc oxide (ZnO), many applications involve this compound in nanostructured form (ZnO-NP). For instance, ZnO-NP has been used as antifungal, antiviral, and antibacterial, or, for its electronic properties, as an efficient component for developing UV fillers or photochemically active materials [14,15]. ZnO has gained more attention than other oxides to obtain nanomaterials as it is considered more eco-friendly and bio-compatible [4]. Moreover, ZnO-NP can be synthesized from its saline precursors applying syntheses that do not involve hazardous chemicals or use high amounts of energy instead of the conventional procedures, which often show a high environmental impact [16].

In this context, the combination of organic nanoparticles, such as lignin nanoparticles (LNP) derived from biomass feedstock, with ZnO-NP to realize nanohybrids can be a suitable strategy to obtain new interesting materials, which show the advantages of the distinct components [7]. In this sense, lignin and zinc oxide (ZnO) hybrid systems represent an organic/inorganic combined nanomaterial, which shows unique structural and electronic properties [13]. Examples of the combined incorporation of lignin and metal oxides in different polymeric matrices can be found in [17,18,19], where the authors found effective results, due to the presence of the hybrids, in terms of enhanced antimicrobial, thermal, and mechanical behavior.

While lignin and ZnO-NP have singly gained attention for numerous agricultural applications, hybrid systems of these materials have gained little attention. Nonetheless, some works have shown the possibility of using lignin nanoparticles to control the release of agrochemicals and fertilizers [20,21]. In particular, nanofertilizers have the advantage of providing specific nutrients, thus strongly limiting their dispersion into the environment. This characteristic allows nanofertilizers to be implemented in precision agriculture that avoids the unnecessary waste of substances compared to classical fertilizers [21]. The same conclusion can be extended to the use of herbicides and fungicides embedded in lignin nanoparticles, which reduces the risk of dispersion of these chemicals into the environment [22,23]. As for ZnO-NP, various positive effects have been found in crops when treated with this material at non-excessive doses [24]. In particular, in relation to the morphology and size of the particles, stimulatory effects on plant nutrition, biomass development, and antioxidant defenses have been recorded [24,25].

In view of the above, the first objective of the present work was the synthesis of a hybrid (ZnO@LNP) composed of lignin and zinc oxide, both in nanoparticle form and its characterization. Subsequently, the effect of the synthesized hybrid was evaluated on maize, which is a globally important crop chosen as a plant model in this study. In particular, the ZnO@LNP was tested on maize using seed-priming technology. Seed nano-priming is considered an effective treatment to induce changes in seed metabolism, positively influencing the early stages of plant development and the whole life cycle [26]. To the best of our knowledge, this is the first study that investigates the effect of a ZnO@LNP hybrid on the seed priming of a crop, exploiting the chemical and physical characteristics of the material, particularly its slow-release action. Furthermore, the application of this material directly to seeds avoids material waste or contains it strongly, making the nano-priming technology effective and sustainable.

## 2. Materials and Methods

### 2.1. Synthesis of LNP, ZnO, and Hybrid ZnO@LNP

LNP suspension was obtained from alkali lignin by hydrochloric acid treatment. Based on the procedures reported in the literature, 4% (*w*/*v*) of alkali lignin in ethylene glycol was maintained under stirring for 2 h at 35 °C [11]. Afterwards, HCl (8 mL, 0.25 M) was mildly added to the solution at a rate of 3–4 drops/min. Then, the suspension was stirred for 2 h and filtered to eliminate soluble impurities from lignin. Afterwards, the solution was dialyzed against deionized water up to neutrality to obtain the LNP suspension.

ZnO-NP have been synthetized by using a procedure already considered in [7]. In detail, 100 mL of NaHCO_3_ 0.15 M were dropped, under ultrasound irradiation for 1 h at 50% of amplitude at 70 °C and under argon flow, to 200 mL of a zinc acetate 0.1 M. The precipitate was centrifuged, washed, and calcined at 350 °C for 1 h.

For the synthesis of hybrid materials, 250 mL of a mixture of lignin (2.76 g), zinc nitrate (0.57 g), and hexamethylenetetramine (0.42 g) was treated with high-power ultrasound for 45 min at 50% of amplitude at 90 °C and under argon flow [7]. After the synthesis, the precipitate was centrifuged, washed, and dried. The high-power ultrasound irradiation was performed using an Ultrasonic processor VCX750 (Sonics & Materials, Inc., Newton, CT, USA), 20 kHz, with a diameter tip of 13 mm.

### 2.2. Characterization of LNP and Hybrid ZnO@LNP

Thermogravimetric measurements (TGA) of nanoparticle powders were performed under airflow (50 mL min^−1^) by using a Seiko Exstar 6300 (Seiko Instruments Inc., Chiba, Japan), applying a heating scan from 30 to 900 °C at 10 °C min^−1^.

Fourier infrared (FTIR) spectra of lignin nanoparticles, ZnO-NP and hybrid ZnO@LNP were recorded using a Jasco FTIR 615 spectrometer (Jasco Corporation, Tokyo, Japan) in the 4000–400 cm^−1^ range, in transmission mode. The materials were analyzed using KBr discs prepared by using pulverized natural materials and KBr powder.

The water contact angle of LNP and ZnO@LNP powders was also evaluated using the sessile drop method. Contact angles of the drops (FTA 1000, First Ten Angstroms, Newark, CA, USA) were measured at room temperature under static conditions on pressed disks of powdered materials; the measurement values represent the mean value of 10 drops. The analysis was estimated by measuring the angle of a 20 µL deionized water sessile drop on the disc surfaces.

Lignin and ZnO hybrid nanoparticles were examined using a field emission scanning electron microscope (FESEM, Zeiss Supra 40, Dresden, Germany) at an operating voltage of 5 kV. Single drops of LNP and ZnO@LNP aqueous suspensions were cast onto a silicon substrate, dried for 24 h, and gold sputtered before the analysis.

Elemental composition and chemical mapping were determined using a Bruker Quantax 200 ED (Bruker, Billerica, MA, USA).

TEM images were obtained using a Philips 208 Transmission Electron Microscope. (Philips, Eindhoven, The Netherlands). The samples were prepared by putting one drop of an ethanol dispersion of the catalyst powder on a copper grid pre-coated with a Formvar film and dried in air.

The XRD patterns were recorded with a Philips X’Pert PRO MPD diffractometer (Philips, Malvern, UK) operating at 40 kV and 40 mA, with a step of 0.03° and a time per step of 30 s using Cu Ka radiation and an X’Celerator detector.

The ICP analyses to determine Zn were performed by using a Varian 700-ES series (Varian, Santa Clara, CA, USA) inductively coupled plasma-optical emission spectrometer (ICP-OES) (Agilent Technologies, Santa Clara, CA, USA) on solutions prepared by dissolving the ZnO@LNP with 2 mL of HNO_3_ 65 wt % and 1 mL of H_2_O_2_ 35% *v*/*v* and adequately diluted.

### 2.3. DPPH Activity of ZnO@LNP

The antiradical activity of lignin and hybrid solutions was tested using a spectroscopic method, based on the disappearance of the absorption band at 517 nm of the free radical 2,2-diphenyl-1-picrylhydrazyl (DPPH) upon reduction by an antiradical compound. The test consisted of adding a certain amount of the aqueous solutions into 2 mL of a DPPH solution in methanol (25 mg mol^−1^ L^–1^) to have 50 mg L^−1^ as the concentration of the LNP solution in methanol. After that, the intensity of the 517 nm absorption band was measured by using an ultraviolet–visible (UV–Vis) spectrophotometer (Varian, Santa Clara, CA, USA). The antioxidant activity was expressed as the ability to scavenge the stable radical DPPH, which was calculated as radical scavenging activity (RSA) using the following equation (Equation (1)):

(1) $$\left(RSA,\;\%\right)=\left[(A_{control}-A_{sample}\right)/A_{control}]\;\times \;100\;$$

where A_control_ and A_sample_ are the absorbances of the control (methanol) at t = 0 min and tested sample after 1 h, respectively.

### 2.4. In Vitro Release Profile of Zn

To quantify the release of Zn^+2^, 100 mg of ZnO@LNP were stirred in 100 mL of water at pH = 7 and 25 °C. After 1, 4, 8, and 24 h, 5 mL of solution were taken, filtered, and analyzed by atomic absorption spectroscopy. The percentage of Zn^+2^ release was calculated as follows (Equation (2)):

(2) $$ZnR=\frac{Amount\;of\;released\;Zn\;x\;100}{\;Total\;amount\;of\;Zn\;in\;ZnO@LNP}.\;$$

### 2.5. Seed Nano-Priming and Measurements

Maize seeds (cv Belgrano) were sterilized with an aqueous solution of NaClO (0.25%, *v*/*v*) for three minutes. Then, seeds were copiously washed with distilled water. To prime seeds, ZnO@LNP suspensions were sonicated for 5 min at the following concentrations, which was expressed as ZnO-NP content in mg for liter:

🗸 Control: only water
🗸 T1: 8 mg of ZnO@LNP containing 0.32 mg of ZnO in water at a final volume of 100 mL to reach a ZnO concentration of 3.2 mg L^−1^;
🗸 T2: 32 mg of ZnO@LNP containing 1.28 mg of ZnO in water at a final volume of 100 mL to reach a ZnO concentration of 12.8 mg L^−1^;
🗸 T3: 128 mg of ZnO@LNP containing 1.28 mg of ZnO in water at a final volume of 100 mL to reach a ZnO concentration of 51.2 mg L^−1^;
🗸 T4: 512 mg of ZnO@LNP containing 20.48 mg of ZnO in water at a final volume of 100 mL to reach the concentration of 204.8 mg L^−1^;
🗸 T5: 2.048 mg of ZnO@LNP containing 81.92 mg of ZnO in water at a final volume of 100 mL to reach the concentration of 819.2 mg L^−1^.

Then, seeds were immersed in 10 mL of these solutions and maintained for 24 h under slow stirring. Afterwards, seeds were placed on paper in Petri dishes (10 seeds/plate), added with 10 mL of distilled water, and maintained in the dark (24 ± 2) °C. The number of germinated seeds, and their radicle length, were recorded at 4 and 5 days after priming, respectively. The germination index was estimated according to Navarro-López et al. [27], using the following equation (Equation (3)):

(3) $$GI=\left(G\;\times \;L\right)/\left(Gw\;\times \;Lw\right)\;$$

where G and L are the germination and radicle length G and L recorded for a specific treatment, and Gw and Lw are the values recorded for control seeds.

The relative seed germination (RSG) was estimated in primed seeds, using the following equation (Equation (4)) [27]:

(4) $$SG=\frac{Seeds\;germinated\;in\;the\;treating\;solutions}{Num\;of\;seeds\;germinated\;in\;water}\;\times \;100.$$

### 2.6. Plant Materials and Growth Conditions

Five days after priming, young maize seedlings, controls, and those treated with T1, T2, T3, and T4 were transferred into hydroponic solutions (24 total seedlings per treatment distributed in 3 trays in order to have three replications). Seeds primed with T5 were excluded due to the clear toxicity exerted by this treatment (Table 1). Then, the samples were grown according to a published procedure [28] in a nutrient solution consisting of 2 mM Ca(NO_3_)_2_ ∙ 4H_2_O, 0.5 mM MgSO_4_ ∙ 7H_2_O, 0.7 mM K_2_SO_4_, 0.1 mM KCl, 0.1 mM KH_2_PO_4_, 1 μM H_3_BO_3_, 0.5 μM MnSO_4_ ∙ H_2_O, 0.5 μM CuSO_4_, 0.5 μM ZnSO_4_ ∙ 7H_2_O, 0.01 μM (NH_4_)_6_Mo_7_O_24_ ∙ 4H_2_O, and 100 μM Fe-EDTA. Seedlings were maintained at (24 ± 2) °C and light intensity of 150 μmol m^−2^ s^−1^ (photoperiod: 12/12 h). After 14 days (second fully developed foliar stage), the plants’ shoot and root length and fresh weight were recorded. Then, the proper amount of plant material was harvested and submitted to the following analyses. 

### 2.7. Estimation of Chlorophyll a and b, Carotenoid, Anthocyanin, Total Phenols, DPPH, MDA

Chlorophyll a and b and carotenoids were assessed in maize seedlings collected 14 days after the priming. First, 1.0 g of leaf samples were extracted with 85% acetone in water (*v*/*v*), with a pestle in a mortar using quartz sand. Then, the suspension was filtered, and the absorbance was recorded at 452.5, 644, and 663 nm to obtain chlorophyll a and b as well as the carotenoid contents [29]. In addition, the total chlorophylls concentration (Chl a + Chl b) was estimated as the sum of Chl a and Chl b, while the relative ratio of these pigments was as expressed as Chl a/Chl b.

As for anthocyanin determination, 0.5 g of fresh maize shoots were collected and extracted with ethanol (95%). The resulting suspension was centrifuged at 5000 rpm for 20 min. The anthocyanin content was determined at 535 and 650 nm [30].

First, 1.0 g of fresh maize shoots were extracted with 10 mL of methanol and left to stir at room temperature overnight to determine the TP and DPPH contents [31]. Afterwards, the suspension was centrifuged at 7000 rpm for 20 min, and the supernatant was filtered. The TP content was determined by adding 0.5 mL of the extracts to 7 mL of distilled water and 0.5 mL of Folin–Ciocalteu’s phenol reagent. After 5 min, 2 mL of 2% sodium carbonate (*w*/*v*) were added to this solution, which was incubated at room temperature for 2 h. Finally, the absorbance was read at 750 nm reporting the TP as mg of gallic acid equivalent (GAE) g^−1^ per dry weight (DW) [31].

The antioxidant activity of the methanolic plant extracts, obtained as described above, was assessed toward the DPPH. First, 0.5 mL of the methanolic extract was added to this scope with 1 mL of ethanol and 1 mL of a solution 0.101 mM of DPPH. Then, the solution so obtained was left to react for 10 min at room temperature. Subsequently, the absorbance was recorded at 517 nm, and the scavenge ability of the extract toward the DPPH was calculated as RSA, as indicated in Section 2.2.

The MDA content was determined in maize subjected to the various treatments and collected 14 days after priming. To this end, 0.5 g of fresh shoot tissue was homogenized with 5% (*w*/*v*) trichloroacetic, and the resulting suspension was centrifuged at 5000 rpm for 15 min. Then, the supernatant was added to 0.5 mL of 0.25% (*w*/*v*) thiobarbituric and heated at 95 °C for 20 min. After quick cooling, the MDA content was quantified spectrophotometrically [28].

### 2.8. Statistical Analysis

Each value reported is the mean of the data from three independent experiments on at least three biological replicates per experiment. Statistical analysis of the data was performed in ANOVA mode by examining the variance with Duncan’s test at *p* < 0.05. The R statistical environment was used to conduct the statistical analysis.

## 3. Results

### 3.1. ZnO@LNP Characterization

**Figure 1:**
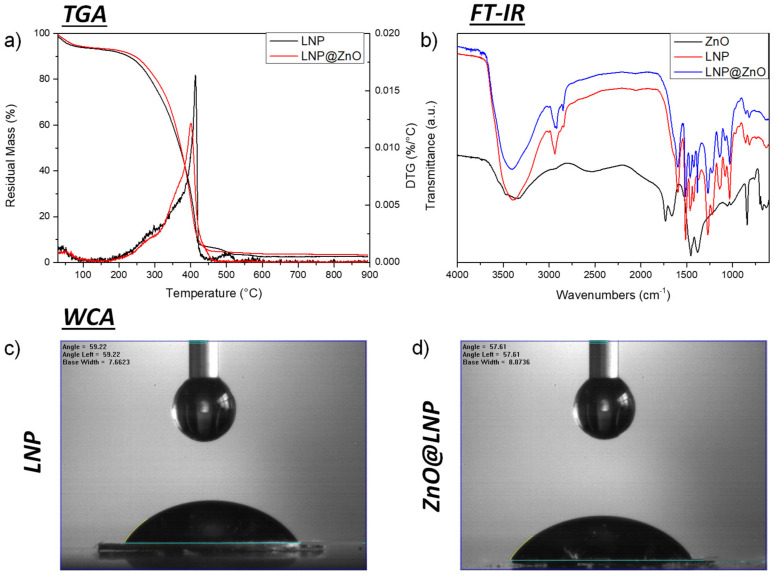
TG/DTG curves of LNP and ZnO@LNP in air (**a**), FTIR spectra of LNP, ZnO, and ZnO@LNP (**b**), water contact angle images for LNP and ZnO@LNP (**c**,**d**).

TGA analysis for the two prepared nanoparticles was performed, and the results of their thermal degradation behavior in the air are reported in Figure 1a. The technique was also used to determine the amount of ZnO on the surface of the hybrid lignin nanoparticles. The results confirmed that lignin decomposition started by water release, which was followed by the first decomposition step between 200 and 300 °C, which led to the formation of low molecular weight products due to propanoid’s side-chain cleavage. The main decomposition step occurred at higher temperatures (300–450 °C), with the cleavage of the main lignin chain, and was followed (above 500 °C) by several rearrangements and condensation reactions of the aromatic structure that leads to the formation of char structures [32,33]. The degradative path of ZnO@LNP was similar, with a slight shift toward higher temperature for the initial degradation events, a lower degradation rate related to the main, but a lower peak temperature (400 and 413 °C, respectively for ZnO@LNP and LNP), which was essentially related to the catalytic role of ZnO-NP nanoparticles at high temperature [34]. The degradation in the oxidative environment (not shown here) confirmed that 4 wt % of ZnO was found on the LNP surface.

FTIR analysis of LNP, ZnO-NP, and ZnO@LNP was also conducted. The analysis results, reported in Figure 1b, showed that in the case of LNP, the typical signals of the nanoscaled lignin already observed in previous investigations were found [11]. In detail, a broad band at 3600–3300 cm^−1^, corresponding to hydroxyl groups in phenolic and carboxylic acids and bands around 2936 and 2840 cm^−1^, attributed to C-H stretch present in methyl, methylene, and methoxyl groups, were found. Signals at 1512 and 1421 cm^−1^, attributed to the aromatic ring vibrations of phenylpropanoid (C9) skeleton of lignin and bands detected at 1270 and 812 cm^−1^, corresponding to C=O stretch in guaiacyl unit and C-H out-of-plane vibrations in 2, 5, and 6 positions of guaiacyl units, were also identified. Furthermore, the spectrum of LNP shows intensity bands at 1216 cm^−1^ (C-C, C-O, and C=O stretching), 1120 cm^−1^ and 1082 cm^−1^, assigned, respectively, to condensed aromatic units and C-O stretch of secondary alcohols and aliphatic ethers. In the case of ZnO, the peaks observed at 3323, 1453, 1380, 1336, 1056, and 1017 cm^−1^ are due to the O-H stretching and bending vibrations of adsorbed water molecules [35]. Additionally, peaks at 1730, 1660, and 1532 cm^−1^ can be identified with the presence of CO_2_. The absorption peak associated with the Zn-O stretching band appears clearly at 500 and 702 cm^−1^, confirming the formation of ZnO-NPs [36]. The peaks at 530 and 411 cm^−1^ can also be assigned to the metal oxygen (M-O) stretching mode [37]. Comparison of the obtained spectra for LNP and ZnO@LNP showed that a reduction of OH stretching can be found at higher wavenumbers, while shifting of bands at 2927, 2918, and 2849 cm^−1^, that represent C-H stretching, and 1451 and 1384 cm^−1^ (ascribed to the in-plane bending vibrations of the CH_2_ groups) were noted. The signal of C-C stretching of aromatic skeletal at 1540 cm^−1^ was also more visible in ZnO@LNP. Narrow peaks appeared near 464, 478, and 505 cm^−1^ represent transverse optical stretching vibrations of Zn-O, confirming the presence of zinc oxide nanoparticles, and these observations were well consistent with other reports [38]. The reduced wettability in the presence of ZnO was also confirmed by the results of water contact angle measurements, as reported in Figure 1c,d. Mean values of 59 and 57° were registered, respectively, for LNP and ZnO@LNP, supporting the changes in surface hydrophilicity for the lignin nanoparticles in the presence of hydrophobic zinc oxide. Variation in surface wettability could also be due to different porosities and roughness of the material.

**Figure 2:**
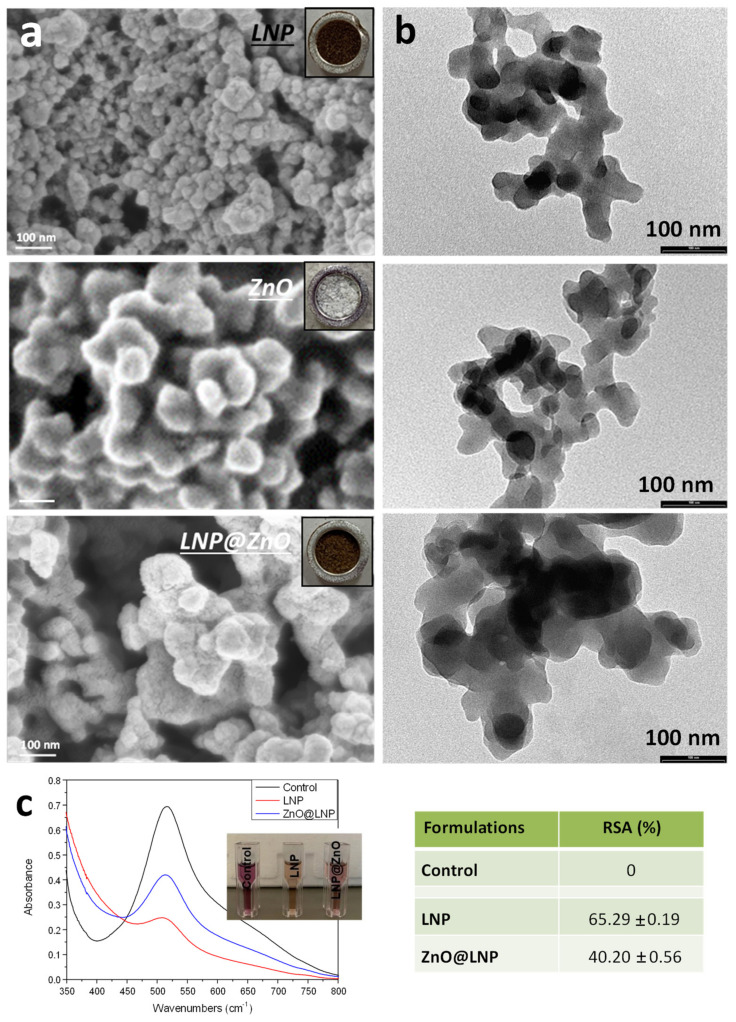
FESEM (**a**) and TEM (**b**) images of LNP, ZnO, and ZnO@LNP (the inset images show the color change due to the formation of lignin-derived ZnO), UV spectra (**c**) and RSA results for LNP and ZnO@LNP.

As shown in Figure 2a, FESEM images of LNP, ZnO, and ZnO@LNP at high magnifications confirmed that the ZnO@LNP sample consists of uniformly dispersed nanosized particles of zinc oxide, with more or less spherical morphology, “encapsulated” on the cavities provided by the macromolecular matrix lignin [39]. The TEM images (Figure 2b) confirm the SEM analyses.

The presence of phenolic groups even on ZnO@LNP (as revealed by FTIR spectroscopy) motivated us to explore their antioxidant potential further. The results of DPPH scavenging activity for pristine LNP and ZnO@LNP systems are reported in Figure 2c. Compared to the control, the absorbance band at 517 nm decreased, while the DPPH scavenging activity increased up to 65.3% after 1 h of incubation for LNP. As widely reported in the literature [10], the antioxidant activity of lignin is determined by the high number of phenolic moieties, which undergo a proton-coupled electron transfer mechanism. Meanwhile, considering the high specific surface area and small spherical lignin nanoparticle size, all of these factors contribute to higher proton capability for the phenyl group of lignin. When ZnO was considered, a sensible reduction in RSA values (40.2%) was measured, suggesting that some phenolic groups were linked to lignin by hydrogen bonds, as confirmed by the shifting for a few bands in FTIR analysis, or they were oxidized during the preparation of the hybrid, which can explain the loss of antioxidant power [40]. The antioxidant property of ZnO@LNP may also be due to a superposition with the electron donation property of oxygen atom in ZnO nanomaterial, as already reported in previous studies [39].

**Figure 3:**
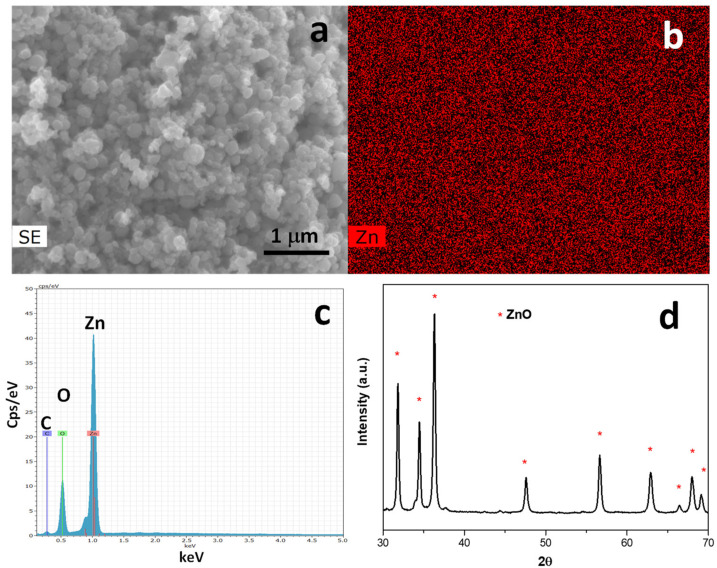
SEM image of the area analyzed by EDX (**a**), elemental mapping of Zn (**b**), EDX spectrum (**c**), and XRD (**d**) of ZnO@LNP.

EDX analyses were performed to verify the homogeneous dispersion of Zn on the LNP surface (Figure 3a–c), while XRD analysis (Figure 3d) showed the characteristic reflection peaks of ZnO: 2θ at 31.8°, 34.4°, 36.3°, 47.5°, 56.7°, 62.8°, 64.4°, 67.9°, and 69° (JPCDS 01-079-2205).

**Figure 4:**
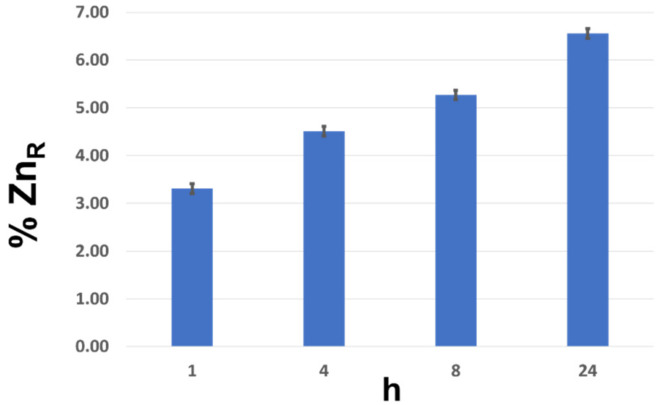
In vitro release profile of Zn^+2^ by ZnO@LNP.

The release profile of Zn^+2^ (Figure 4) shows that after 1 h, the percentage of Zn released in solution was 3.3%, and as the time increased, after 24 h, it reached the value of about 6.5%. The release test highlighted the material’s ability to slow-release Zn^+2^ over time, and this phenomenon could demonstrate the ability of the ZnO@LNP to release zinc when in contact with maize seeds. Therefore, such an effect can evidence the hybrid’s capacity to stimulate the growth and development of the plant.

### 3.2. Effect of ZnO@LNP on Seed Development and Maize Growth

The nano-priming with ZnO@LNP affected seed germination, radicle length, RSG, and GI (Table 1). In particular, T2 and T3 stimulated the seed germination significantly compared to control seeds. The other treatments did not influence this parameter, except for T5, which depressed the capacity of seeds to germinate. Data of relative seed germination (RSG) revealed that T2 and T3 significantly impacted this parameter. The germination represents a critical phase in plant development, and effective seed priming has been reported to positively affect seed metabolism and promote numerous signaling pathways, thus improving seedlings establishment and the entire lifecycle of the primed plants [26]. Some nanomaterials can positively affect seed development if the dosage applied does not reach phytotoxic levels. Furthermore, seed nano-priming could stimulate the expression of many genes during germination, especially those related to plant stress tolerance [41,42]. This last aspect is crucial, as improving plant tolerance to abiotic and biotic stress through seed priming could be strategic for crop protection [42]. Seed germination is mainly regulated by signals that include the controlled production of reactive oxygen species (ROS), resulting in improved water absorption and cell extension [43]. In this context, zinc is involved in the controlled production of ROS and the activation of NADPH oxidase, α-amylase, and protease. Furthermore, Zn can induce the production of secondary metabolites, which can positively affect the first stage of seeds’ development [44,45]. In particular, seed attributes can be improved by ZnO-NP for its electronic characteristics, which can give rise to the photosensitization and photogeneration of ROS [46]. Consequently, this improves oxygen imbibition and water absorption by seeds, thus allowing faster and ameliorated seed germination [47]. Despite this, excessive concentrations of nanomaterials can hamper seed metabolism and development and consequently plant growth, thus generating a plethora of phytotoxic effects in plants [48,49]. For this, the controlled release of ZnO-NP through nanocarrier delivery appears to be a strategic approach to avoid an immediate exposure of seeds to excessive concentrations of this oxide [44]. The use of lignin nanoparticles as a nanocarrier for the controlled release of ZnO-NP could permit the achievement of noteworthy results on seed development without exerting toxicity on a wide range of concentrations. Moreover, it should be considered that lignin is a biocompatible polymer that is relatively biodegradable, which makes its use safe. In addition, lignin itself is known to promote beneficial effects on plant growth as it can influence the hormonal status and the development of seeds [11].

Radicle length, recorded five days after the treatments, revealed that the nano-priming generally stimulated radicle length, while T5 was the only phytotoxic treatment. Furthermore, the germination index (GI), which combines the effects of the priming solution on seed and radicle length (for this measure, the control is considered as 100%), revealed that the treatment positively affected this parameter, even though to a different extent, with the only exception of T5. Zinc regulates hormone metabolism, which controls the first phases of seed and radicle development by affecting the auxin levels [47]. This effect occurs thanks to the ability of this zinc to modulate the tryptophan biosynthesis [47]. In general, it has been found that depending on the treated species, ZnO-NP can result in different effects on radicle elongation, also causing severe toxic effects [50]. Nonetheless, hybrid nanoparticles can positively affect germination and root length, and this action has been explained on the efficacy of the nanosystems to allow easier penetration of the seed coat and increase the number of holes [26]. Such an effect may also result in increased oxygen transfer to seeds and water uptake efficiency [51,52].

### 3.3. Effect of ZnO@LNP on Plant Growth

**Figure 5:**
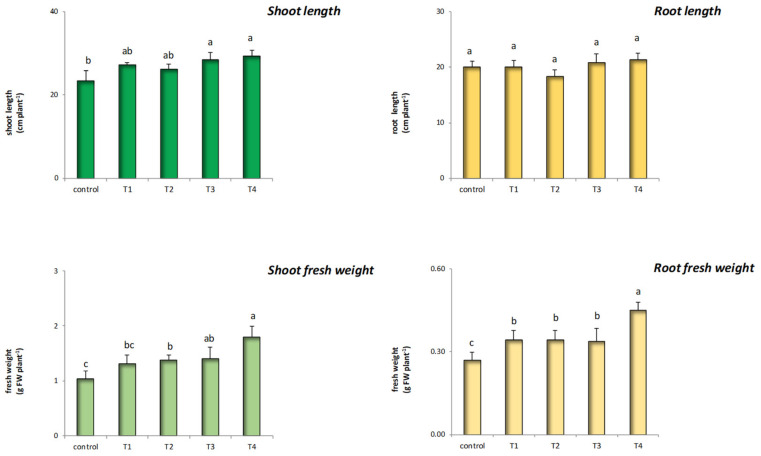
Effect of the treatments with ZnO@LNP on the shoot and root length and fresh weight compared to the untreated controls (T1, T2, T3, T4, and T5 refer to the ZnO@LNP concentration used for seed nano-priming). Letters in the figure, if different, indicate statistically significant differences for *p* < 0.05 between treatments.

Since the results obtained from the above determinations showed that T5 was phytotoxic to maize, this treatment was not further included in the subsequent determinations (Table 1). As far as shoot length is concerned, T3 and T4 increased this parameter in nano-primed maize, while the other treatments did not show any effect (Figure 5). Differently, all the ZnO@LNP concentrations applied did not affect root length (Figure 5). The shoot fresh weight was stimulated by T2, T3, and T4, whilst T1 did not affect maize compared to control samples. As for root fresh weight, all the treatments stimulated this trait on treated plants, with T4 showing a remarkable effect. Priming with nanomaterials may also benefit seedlings by stimulating plant growth and defensive mechanisms, which were mainly related to increased antioxidant properties [46]. The mechanism by which ZnO-NP activates these processes is still largely unknown and under debate; however, several studies have shown that this material can improve the performance of the treated species at low dosages, increasing root and shoot biomass [46]. The effect of ZnO-NPs, in addition to the concentrations applied, was related to the mode of its application. In particular, direct administration to the growth medium in developing plants can decrease their growth due to rapid dissolution of the oxide in Zn^2+^, which could alter already at low concentrations root epidermis, thus compromising the entire functionality of the root organs [24,53]. Nonetheless, it has been shown that nanoparticle systems, in general, can activate the expression of genes involved in nutrient transport in plants [24]. Moreover, it should be noted that the use of nanocarriers has the advantage of allowing a controlled release of zinc oxide, significantly reducing its toxicity and maximizing its benefits [44].

### 3.4. Effect of ZnO@LNP on Pigment and Soluble Protein

Some other aspects of nano-primed plants were investigated, and they are shown in Table 2. In particular, the content of chlorophyll a and b (Chl a and Chl b), the sum (Chl a + Chl b), the ratio Chl a/Chl b, and the carotenoid content showed trends generally in agreement with each other. As for Chl a, the treatments T2, T3, and T4 affected the pigment content in nano-primed maize, while T1 did not. Concerning Chl b, none of the treatments applied modified its content. Regarding the sum of Chl a and Chl b, T2, T3, and T4 determined a significant increase in the total amount of chlorophylls. Some work showed that nanomaterials, mainly metal oxide nanoparticles, can affect the content of chlorophyll a and b, also in abiotic stress conditions such as salinity and heavy metals contamination [54,55,56,57]. Zinc participates in chlorophyll synthesis, being involved in protochlorophyllide formation and chloroplast development [46]. This biochemical aspect is relevant, since the effect of ZnO@LNP resulted in significant increases in chlorophyll a and total chlorophyll. This stimulation reveals that ZnO@LNP treatments may prompt higher photosynthetic activity, resulting in increases in plant biomass production, justifying the data obtained in this study (Figure 5). In addition, increases in chlorophyll a are associated with the physiological adaptation of plants to external stimuli aimed to increase the plant’s capacity to harvest light [57]. The ratio Chl a/Chl b, usually around 3, can be predictive of the amount of chlorophyll associated with the photosystems, and, also, it is used to estimate plant responses to environmental changes and stress or external stimuli [58]. Our experiments showed significant increases in this ratio only in the case of T2 and T3. This effect is worth mentioning, as the chlorophyll–protein complex, which has the function to harvest light, is positively related to the activity of photosystem II (PSII) [58]. Therefore, the results show that ZnO@LNP can be a valuable tool to stimulate the content of chlorophyll a and the activity of PSII.

The carotenoid content increased in response to T2, T3, and T4, while T1 did not affect this parameter. Carotenoids are essential light-harvesting pigments with protective functions. They show antioxidant properties and operate in removing ROS, thus protecting chloroplast from oxidative stress that biotic and abiotic stresses could cause [59]. In addition, carotenoids operate in quenching chlorophyll in triplet or singlet form [46]. Carotenoids are gaining increasing interest for their nutraceutical value as bioactive and health-promoting compounds, and there is intense research toward strategies that can raise the content of molecules with known nutraceutical value [60]. As mentioned above, zinc can stimulate the activity of NADH oxidase and the formation of ROS, and this effect persists during the later stages of development of the treated plant [44,45]. Therefore, the plant response associated with the treatment with ZnO@LNP was that of elevating the content of this beneficial and pivotal class of protective pigment, as evidenced by other studies [46,61].

### 3.5. Effect of ZnO@LNP on Antioxidants and Oxidative Status of Nano-Primed Maize Seedlings

Some determinations have been conducted on seeds nano-primed with ZnO@LNP to evidence any eventual effect on the nanomaterial on the antioxidant and oxidative status of seedlings collected 14 days after the treatments (Table 3). T2, T3, and T4 generally elevated the anthocyanin and TP content compared to control samples, while T1 was unable to exert an inductive effect on the above parameters.

Many different secondary metabolites show scavenging activities against free radical and antioxidant properties, and their content in plants can be modulated in response to environmental factors [62]. In particular, anthocyanins have a protective function in the vacuole by inhibiting or containing the lipid peroxidation, and their production could increase as a photo-protection to avoid photo-oxidative injuries [46]. Phenols have been documented to increase the following abiotic stresses such as salinity, drought, heavy metals, and others, as they can directly remove ROS or chelate metals [60]. However, phenolic metabolites are health-promoting compounds with therapeutic potential against many human diseases. For this reason, finding agents that could enhance the production by plants of secondary metabolites is not only an improved attribute for the plant itself, but it deserves attention as it can improve the nutraceutical values of crops [63,64]. In this context, our results revealed interesting increases in the content of anthocyanin and TP at the highest dosages applied for seed nano-priming (T2, T3, and T4). This effect has been documented in plants exposed to ZnO-NP, which, for the properties of its valence electrons, can absorb close to 400 nm and promote photo-oxidative processes [65]. In particular, ZnO-NP show an intense absorption in the range of 370–390 nm, depending on the nanoparticles dimensions, size, and surface [46]. As for phenols, the mechanisms activated in response to nanoparticle treatment are largely unknown, although some experimental evidence indicates that ZnO-NPs may regulate phenols synthesis at the transcriptional level [64]. In addition, ZnO-NP can give rise to ROS production, which may increase the antioxidants and secondary metabolites [66]. Nevertheless, due to the protective role of phenols in plants and their effect on their qualitative profile of crops, an increase in their content deserves attention.

Finally, the DPPH antioxidant activity and MDA content in nano-primed seedlings with T1, T2, T3, and T4 were determined (Table 3). The DPPH activity is routinely assessed to evidence the antioxidant activity following the exposure to abiotic and biotic stress or the effect of different treatments [67]. The results obtained in this study are consistent, since what was observed for carotenoids, anthocyanin, and TP aligned with the increases in the DPPH activity following the treatment with ZnO@LNP. Therefore, it can be reasonably supposed that the stimulatory effect of the hybrid regarded mainly the antioxidant activities thanks to the documented ability of ZnO-NP to give rise to oxidative perturbations, that, however, if reaching sub-toxic levels can promote beneficial effects on treated plants [46,67].

The last aspect investigated in this study was the content of malondialdehyde (MDA), which is a product of lipid peroxidation. Results evidenced that nano-primed maize with T2, T3, and T4 significantly decreased the MDA compared to control samples. This result is indicative of the effectiveness of ZnO@LNP treatments in potentiating the antioxidant responses in primed seeds. MDA is commonly produced in chloroplasts and mitochondria, where metabolic processes characterized by high electron flow occur [28,68]. In stressful situations, usually, plants increase the content of molecules and enzymes with antioxidant action [63]. MDA accumulates when oxidative stress is particularly severe, and antioxidant defenses fail to contain ROS [46]. Therefore, the MDA estimation becomes important, as it indicates the cell’s oxidative state. The results obtained in this study agree with the profile of the antioxidants investigated (Table 2 and Table 3) and shed light on the capacity of the ZnO@LNP to ameliorate the oxidative status of nano-primed seeds. Other studies have attributed such a beneficial effect on lipid peroxidation containment to the capacity of nanomaterials to stimulate antioxidant activities [29,48]. These aspects should receive attention as the treatment with nanomaterials by foliar application or nano-priming can also be a strategy to prepare plants to cope with abiotic or biotic stresses by improving their antioxidant defenses.

## 4. Conclusions

The use of nanomaterials as nanocarriers is gaining attention in agriculture, where they have recently been introduced. These new nanosystems stimulate plant growth and development, opening new perspectives in using nanoparticles [69]. The objective of this work was to realize a hybrid consisting of lignin and ZnO, both in nanoparticle form. The hybrid function was to deliver and release bioactive zinc oxide nanoparticles to plants with the scope of biofortifying the treated species. To this end, after the hybrid synthesis, ZnO@LNP were applied at different concentrations using nano-priming technology. The beneficial effects recorded on maize at certain concentrations offer encouraging prospects. However, further studies are needed to understand and elucidate how these hybrid systems can exert these effects.

## Figures and Tables

**Table 1:** Effect of the treatments with ZnO@LNP on germination, radicle length, relative seed germination (RSG), and germination index (GI) of maize compared to the untreated controls (T1, T2, T3, T4, and T5 refer to ZnO-LNP concentration used for seed nano-priming).

	Germination	RSG	Radicle Length	GI
	(%)	(%)	(cm)	(%)
Control	77 ± 5 ^b^	-	2.10 ± 0.30 ^b^	-
T1	83 ± 10 ^ab^	109 ± 7 ^b^	2.56 ± 0.50 ^ab^	133 ± 9 ^c^
T2	97 ± 10 ^a^	126 ± 2 ^a^	2.83 ± 0.28 ^a^	170 ± 3 ^a^
T3	93 ± 6 ^a^	122 ± 5 ^a^	2.80 ± 0.27 ^a^	165 ± 6 ^a^
T4	87 ± 8 ^ab^	113 ± 1 ^b^	2.81 ± 0.25 ^a^	151 ± 2 ^b^
T5	56 ± 5 ^c^	54 ± 10 ^c^	1.67 ± 0.40 ^b^	49 ± 9 ^d^

In each column and for each parameter, mean values followed by different letters are significantly different (*p* < 0.05).

**Table 2:** Effects of the nano-priming treatments with different concentrations of ZnO@LNP on the content of chlorophylls a and b, their sum and relative ratio, carotenoids, and soluble protein (T1, T2, T3, T4, and T5 refer to ZnO@LNP concentration used for seed nano-priming).

	Chl a	Chl b	Chl a + Chl b	Chl a/Chl b	Carotenoids
	(mg g^−1^ FW)	(mg g^−1^ FW)	(mg g^−1^ FW)		(mg g^−1^ FW)
Control	3.88 ± 0.30 ^b^	1.16 ± 0.05 ^b^	5.04 ± 0.28 ^b^	3.34 ± 0.34 ^b^	0.35 ± 0.02 ^b^
T1	3.99 ± 0.22 ^b^	1.09 ± 0.14 ^b^	5.08 ± 0.35 ^b^	3.66 ± 0.30 ^ab^	0.33 ± 0.04 ^b^
T2	4.62 ± 0.24 ^a^	1.19 ± 0.06 ^b^	5.81 ± 0.31 ^a^	3.88 ± 0.11 ^a^	0.41 ± 0.03 ^a^
T3	4.86 ± 0.41 ^a^	1.17 ± 0.22 ^b^	6.03 ± 0.50 ^a^	4.15 ± 0.61 ^a^	0.44 ± 0.01 ^a^
T4	4.63 ± 0.28 ^a^	1.26 ± 0.28 ^b^	5.89 ± 0.32 ^a^	3.67 ± 0.11 ^b^	0.41 ± 0.03 ^a^

In each column and for each parameter, mean values followed by different letters are significantly different (*p* < 0.05).

**Table 3:** Effects of the nano-priming treatments with different concentrations of ZnO@LNP on the content of anthocyanin, total phenols (TP), DPPH, and MDA (T1, T2, T3, T4, and T5 refer to ZnO@LNP concentration used for seed nano-priming).

	Anthocyanin	TP (mg g^−1^ FW)	DPPH	MDA
	(mg g^−1^ FW)	(Gallic Acid Equivalents)	(Scavenging Rate %)	(nmol g^−1^ FW)
Control	0.14 ± 0.01 ^c^	0.76 ± 0.04 ^c^	65.3 ± 1.6 ^b^	12.2 ± 1.0 ^a^
T1	0.16 ± 0.03 ^abc^	0.79 ± 0.02 ^c^	72.8 ± 2.9 ^a^	13.1 ± 1.2 ^a^
T2	0.17 ± 0.01 ^ab^	0.86 ± 0.02 ^b^	74.1 ± 1.3 ^a^	10.1 ± 0.7 ^b^
T3	0.18 ± 0.01 ^ab^	0.85 ± 0.04 ^b^	73.9 ± 0.9 ^a^	9.2 ± 0.6 ^bc^
T4	0.20 ± 0.02 ^a^	0.95 ± 0.02 ^a^	76.4 ± 1.0 ^a^	8.8 ± 0.6 ^c^

In each column and for each parameter, mean values followed by different letters are significantly different (*p* < 0.05).
